# Aqueous Extracts of *Ocimum gratissimum* Sensitize Hepatocellular Carcinoma Cells to Cisplatin through BRCA1 Inhibition

**DOI:** 10.3390/ijms25158424

**Published:** 2024-08-01

**Authors:** Jing-Huei Chen, Tsai-Hui Lin, Yu-Chuan Chien, Chung-Yu Chen, Chih-Tung Lin, Wei-Wen Kuo, Wei-Chao Chang

**Affiliations:** 1 Graduate Institute of Biomedical Sciences, China Medical University, Taichung 404333, Taiwan; backeract3j@gmail.com (J.-H.C.); yuchuan0916@gmail.com (Y.-C.C.); 2 Department of Chinese Medicine, China Medical University Hospital, Taichung 404327, Taiwan; 010178@tool.caaumed.org.tw; 3 Research Center for Cancer Biology, China Medical University, Taichung 406040, Taiwan; okada8@gmail.com (C.-Y.C.); 4a1h0037@stust.edu.tw (C.-T.L.); 4 Program for Biotechnology Industry, China Medical University, Taichung 406040, Taiwan; 5 Center for Molecular Medicine, China Medical University Hospital, Taichung 406040, Taiwan

**Keywords:** hepatocellular carcinoma (HCC), *Ocimum gatissimum* aqueous extract (OGE), cisplatin, breast cancer type 1 susceptibility protein (BRCA1)

## Abstract

*Ocimum gratissimum* (*O. gratissimum*), a medicinal herb with antifungal and antiviral activities, has been found to prevent liver injury and liver fibrosis and induce apoptosis in hepatocellular carcinoma (HCC) cells. In this study, we evaluated the effect of aqueous extracts of *O. gratissimum* (OGE) on improving the efficacy of chemotherapeutic drugs in HCC cells. Proteomic identification and functional assays were used to uncover the critical molecules responsible for OGE-induced sensitization mechanisms. The antitumor activity of OGE in combination with a chemotherapeutic drug was evaluated in a mouse orthotopic tumor model, and serum biochemical tests were further utilized to validate liver function. OGE sensitized HCC cells to the chemotherapeutic drug cisplatin. Proteomic analysis and Western blotting validation revealed the sensitization effect of OGE, likely achieved through the inhibition of breast cancer type 1 susceptibility protein (BRCA1). Mechanically, OGE treatment resulted in BRCA1 protein instability and increased proteasomal degradation, thereby synergistically increasing cisplatin-induced DNA damage. Moreover, OGE effectively inhibited cell migration and invasion, modulated epithelial-to-mesenchymal transition (EMT), and impaired stemness properties in HCC cells. The combinatorial use of OGE enhanced the efficacy of cisplatin and potentially restored liver function in a mouse orthotopic tumor model. Our findings may provide an alternate approach to improving chemotherapy efficacy in HCC.

## 1. Introduction

Globally, hepatocellular carcinoma (HCC) is the fifth most common malignant cancer, with more than 900,000 new cases and 830,000 deaths annually [1]. HCC treatment can be divided into two categories: curative therapy and palliative care, depending on disease stage, tumor size, and the availability of a healthy donor liver. Potentially curative treatments include surgical resection, liver transplantation, and local ablation therapy, which can result in 5-year overall survival rates of over 70% [2]. However, less than 30% of patients with HCC can benefit from these treatments [3]. Hepatic artery chemoembolization and targeting therapy are generally used for palliative care in locally advanced or metastatic HCC [4,5]. Despite broad usage in the treatment of various cancers, chemotherapeutic drugs have limited or no benefit in HCC due to its drug-resistant nature [6]. HCC is highly heterogeneous, with tumors containing a variety of cell types with different genetic and epigenetic profiles. The drug-resistant characteristics of HCC cells include epigenetic modifications such as DNA methylation and histone modifications, hyperactivation of the PI3K/AKT/mTOR signaling pathway, high expression of multidrug resistance proteins, and dysfunction of drug metabolism enzymes [7]. Additionally, a small subset of cancer stem cells (CSCs) with tumor-initiating and self-renewal abilities contribute to tumor proliferation, metastasis, and drug resistance [8]. More importantly, systemic chemotherapy is usually not well tolerated by HCC patients with hepatic dysfunction [9]. Therefore, developing chemo-sensitization strategies may provide these patients with more treatment options.

**Figure 1:**
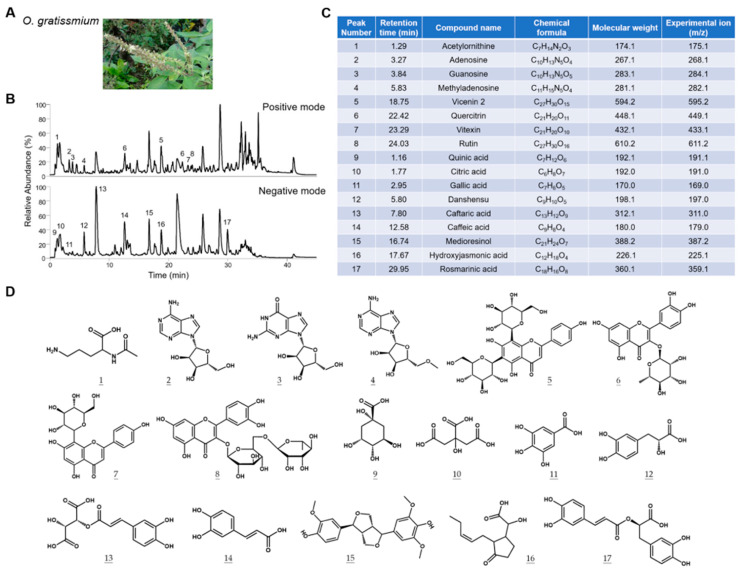
Characterization of the chemical composition of OGE. (**A**) Representative picture of *O. gratissimum*. (**B**) The TIC profiles of OGE in positive ion mode (upper) and negative ion mode (lower). (**C**) The composition of OGE was identified via Compound Discoverer analysis. The corresponding peak numbers and retention times are indicated in (**B**). (**D**) The chemical structures of compounds are identified at each peak.

Herbal medicines have been used for thousands of years in Asian countries, and their application is expanding globally [10]. Historically, herbal compounds have been considered abundant resources of antitumor agents, with more than 60% of pharmaceutical antitumor drugs derived from natural compounds [11]. Due to the potency of herbal compounds in reducing chemoresistance and increasing the efficacy of chemotherapeutic drugs, several clinical trials have been conducted to assess the sensitization effects of herbal compounds in cancers [12]. *Ocimum gratissimum* (*O. gratissimum*) (Figure 1A) is one of the principal species in the *Ocimum* genus, the largest genus in the Lamiaceae family. *O. gratissimum* is a medicinal herb with numerous pharmacological effects, including antifungal, antiviral, anti-inflammatory, and antitumor activities, and it is involved in modulating the immune system [13,14]. The leaves and tea products of *O. gratissimum* are classified as food-grade ingredients in Taiwan (Supplementary Table S1). Our previous studies revealed that aqueous extracts of *O. gratissimum* (OGE) can not only prevent liver injury and liver fibrosis but also induce cellular apoptosis in HCC cells both in vitro and in vivo [15,16,17]. These findings suggest the potential use of OGE as an effective chemosensitizer for HCC treatment.

In this study, we test the hypothesis that OGE could serve as a chemosensitizer for HCC treatment. We evaluate the sensitization effects of OGE and explore the underlying molecular mechanisms. Furthermore, we verify the efficacy of OGE in combination treatment using a mouse model.

## 2. Results

### 2.1. Characterization of the Chemical Composition of OGE

It is well recognized that the extracted chemical components of medicinal herbs can be affected by the source of herbs and extraction methods. To establish the correlation between the composition of OGE and its biological activities, we characterized the components of OGE using ultrahigh-performance liquid chromatography coupled with mass spectrometry (UHPLC-MS) [18]. The total ion chromatogram (TIC) profiles of OGE were determined using Q-Exactive Plus mass spectrometer equipped with the UltiMate 3000 UHPLC system (Thermo Scientific; San Jose, CA, USA) in both positive ion mode (Figure 1B, upper) and negative ion mode (Figure 1B, lower). Precise molecular weight and tandem mass were used for molecular identification with Compound Discoverer software version 3.3 SP2 (Thermo Scientific; San Jose, CA, USA). Seventeen compounds were identified in this analysis (Figure 1C). The chemical structures of the identified compounds are shown in Figure 1D. On the basis of these results, we standardized the composition of OGE according to the relative TIC profiles and molecular composition for subsequent biological assays.

### 2.2. OGE Sensitizes HCC Cells to Cisplatin

**Figure 2:**
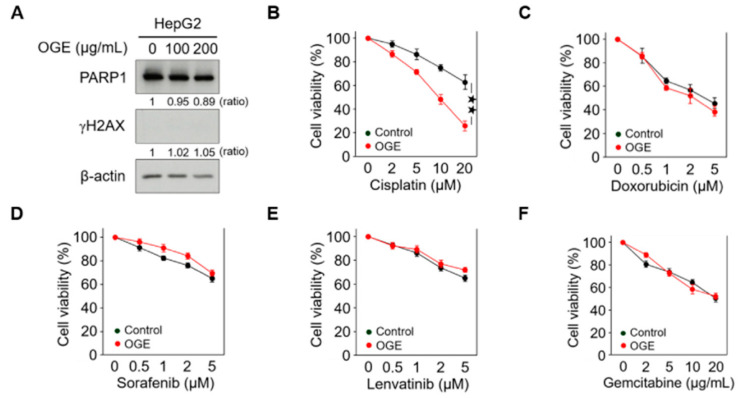
OGE sensitizes HCC cells to cisplatin. (**A**) The expression of PARP1 and γH2AX in HepG2 cells treated with the indicated doses of OGE was determined via Western blotting. Ratio, the relative protein level. β-actin, loading control. (**B**–**F**) HepG2 cells were pretreated or untreated (control) with OGE (200 μg/mL) for 2 h, then treated with the indicated doses of cisplatin (**B**), doxorubicin (**C**), sorafenib (**D**), lenvatinib (**E**), and gemcitabine (**F**) for an additional 24 h. Cell viability was determined using the MTT assay. Data are shown as the means ± SD. Two-tailed unpaired Student’s *t*-test (**B**–**F**). ^★★^, *p* < 0.01.

High doses of OGE showed a cytotoxic effect on HCC cells in our previous study [17]. To evaluate the adjuvant role of OGE in chemotherapeutic drug treatment, relatively lower doses of OGE were used to treat the HepG2 cell line. Western blotting revealed that 100 μg/mL and 200 μg/mL of OGE did not cause significant degradation of poly (ADP-ribose) polymerase 1 (PARP1), a marker of apoptosis, nor did they induce detectable expression of phosphorylated histone H2AX (γH2AX), a marker of DNA damage (Figure 2A). Accordingly, the adjuvant effect of OGE at a dose of 200 μg/mL was evaluated with several chemotherapeutic drugs recommended for HCC treatment, including cisplatin, doxorubicin, sorafenib, lenvatinib, and gemcitabine [19,20,21]. Except for cisplatin (Figure 2B), the adjuvant effect of OGE on other drugs could not be detected using the cell viability MTT assay (Figure 2C–F). Therefore, in this study, we explored the effect of OGE on sensitizing HCC cells to cisplatin.

### 2.3. OGE Suppresses the Expression of BRCA1 in HCC Cells

**Figure 3:**
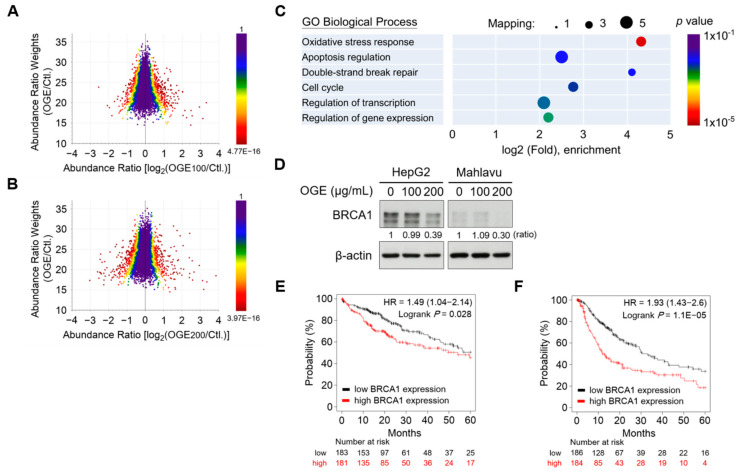
OGE suppresses BRCA1 expression in HCC cells. (**A**,**B**) The three-dimensional scatterplot shows the relationship between abundance ratio weights and abundance ratios for individual identified proteins in the proteomic analysis of OGE (100 μg/mL) vs. untreated control (**A**) and OGE (200 μg/mL) vs. untreated control (**B**). The color represents the *p*-value of an individual protein. (**C**) GO biological process annotation of differentially down-regulatory proteins. The circle size indicates the number of mapping proteins in each process, and the color indicates the *p*-value of each process. (**D**) The expression of BRCA1 in both HepG2 and Mahlavu cells treated with the indicated doses of OGE was determined via Western blotting. Ratio, the relative protein level. β-actin, loading control. (**E**,**F**) The correlation between the expression of BRCA1 and OS (**E**) or PFS (**F**) of patients with HCC was analyzed using the Kaplan-Meier-plotter cancer database. The high versus low expression levels of BRCA1 mRNA were split by the median value. The follow-up threshold for patients was set as 60 months. Statistical analysis, log-rank test (**E**,**F**).

To identify the critical molecules responsible for cisplatin sensitization, we conducted a proteomic analysis to determine the alterations in the protein signature of HCC cells under OGE treatment. HepG2 cells were treated with 100 μg/mL and 200 μg/mL of OGE, respectively, and a total of 5822 proteins were identified in this experiment (Supplementary Table S4). Proteomic alterations in HepG2 cells in response to individual OGE treatments were visualized on a three-dimensional scatterplot (Figure 3A,B). The scatterplot was plotted in terms of the abundance ratio weights (weighting by mass intensity) and abundance ratios of each protein, with the color of the protein dots representing the *p*-value for abundance ratios adjusted via a background *t*-test. The shift of the central line in the profile distribution towards the negative abundance ratio direction suggested that compared to a lower dose of OGE (100 μg/mL), a higher dose of OGE (200 μg/mL) led to greater down-regulation of protein expression (Figure 3A,B).

GO biological process annotation of differentially down-regulatory proteins revealed that OGE significantly suppressed the expression of proteins involved in oxidative stress response, apoptosis regulation, double-strand DNA break repair, and cell cycle regulation (Figure 3C). The proteins significantly down-regulated by OGE were filtered using the following criteria: abundance ratio OGE100/Ctl < 0.4, abundance ratio OGE100/Ctl ≤ OGE200/Ctl, and abundance ratio adj. *p*-value < 0.05 (Table 1 and Supplementary Table S5). Notably, the proteomic results revealed that OGE led to the significant down-regulation of breast cancer type 1 susceptibility protein (BRCA1) in HepG2 cells, which was confirmed by subsequent Western blotting validation (Figure 3D). BRCA1 is frequently mutated in breast and ovarian cancers and plays important roles in multiple processes such as transcription, cell cycle progression, and DNA damage repair [22]. Recently, BRCA1 was reported to serve as a prognostic indicator in HCC cells [23]. Consistent with this finding, in silico Kaplan-Meier survival analysis revealed a significant correlation between higher BRCA1 gene levels and shorter overall survival (OS) and progression-free survival (PFS) in patients with HCC (Figure 3E,F). Collectively, our findings suggest that OGE enhances the efficacy of cisplatin for HCC treatment, which may be related to the regulation of BRCA1.

### 2.4. OGE Increases BRCA1 Protein Instability via Activation of Proteasomal Degradation

**Figure 4:**
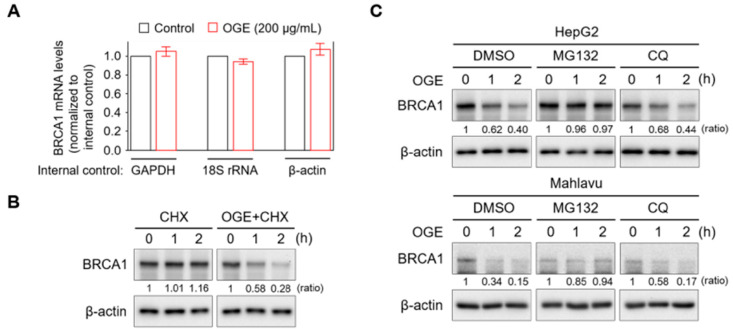
OGE activates the proteasomal degradation of BRCA1. (**A**) BRCA1 mRNA levels in HepG2 cells treated or untreated with OGE (200 μg/mL) for 24 h were determined using qPCR. GAPDH, 18S rRNA, and β-actin served as internal controls for gene expression. (**B**) After cycloheximide (CHX, 10 g/L) reaction for 1 h, HepG2 cells were treated or untreated with OGE (200 μg/mL) for the indicated times. BRCA1 expression in each experimental group was determined via Western blotting. (**C**) After reaction with MG132 (1 nM) or CQ (10 μM) for 30 min, HepG2 and Mahlavu cells were treated or untreated with OGE (200 μg/mL) for the indicated times. BRCA1 expression in each experimental group was determined via Western blotting. Ratio, the relative protein level. β-actin, loading control. Data are shown as the means ± SD. Statistical analysis, two-tailed unpaired Student’s *t*-test (**A**).

To understand how OGE suppresses BRCA1 expression, BRCA1 mRNA levels in HepG2 cells with or without OGE treatment were measured using qPCR. OGE treatment did not exhibit a significant effect on BRCA1 mRNA levels (Figure 4A). However, the pulse-chase experiment showed that BRCA1 protein stability was dramatically reduced by OGE treatment (Figure 4B). Cellular protein degradation primarily relies on both the ubiquitin-proteasome and autophagy-lysosome pathways [24]. To determine which pathway is involved in OGE-induced BRCA1 degradation, MG132 and chloroquine (CQ) were used to inhibit proteasomal and autophagic protein degradation, respectively. CQ was incapable of reducing OGE-induced BRCA1 degradation, while MG132 stabilized BRCA1 under OGE treatment (Figure 4C), suggesting that OGE induces BRCA1 degradation via a proteasomal-dependent pathway.

### 2.5. OGE Enhances Cisplatin-Induced DNA Damage

**Figure 5:**
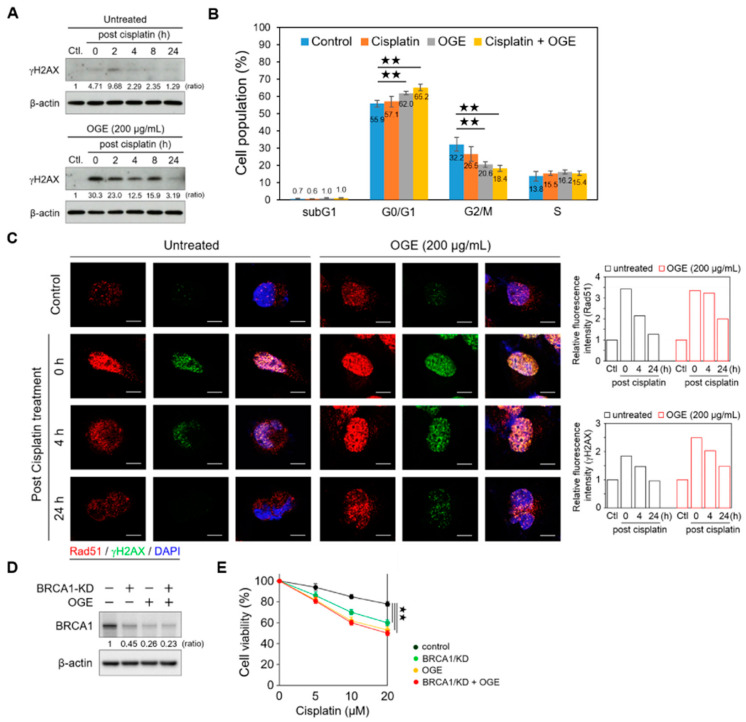
OGE enhances cisplatin-induced DNA damage. (**A**) HepG2 cells, in the presence or absence of OGE (200 μg/mL), were treated with cisplatin (60 μM) for 4 h. After cisplatin removal (defined as 0 h post cisplatin treatment), HepG2 cells were collected at the indicated times. The expression of γH2AX was determined via Western blotting. Ratio, the relative protein level. β-actin, loading control. (**B**) After cell synchronization by culture with serum-free media for 24 h, HepG2 cells, in the presence or absence of OGE (200 μg/mL), were treated with cisplatin (60 μM) for 6 h. The cell cycle was determined using flow cytometric analysis of propidium iodide-stained cells with a FACSVerse flow cytometer (BD Biosciences). Cell cycle distribution was analyzed using BD FACSuite software (BD Biosciences). (*n* = 3). (**C**) HepG2 cells, in the presence or absence of OGE (200 μg/mL), were treated with cisplatin (60 μM) for 4 h. The expression of γH2AX (green) and Rad51 (red) in HepG2 cells at the indicated times post-cisplatin treatment was analyzed using confocal microscopy. The fluorescence signals were measured using ImageJ 1.54g software, and the relative fluorescence intensity was normalized to the control group. (**D**) BRCA1 expression was determined via Western blotting, and (**E**) cell viability assays were conducted using the MTT assay in HepG2 cells with or without BRCA1/KD in the presence or absence of OGE (200 μg/mL) at the indicated doses of cisplatin. The nuclei, DAPI (blue). Scale bar, 10 µm. For statistical analyses, one-way ANOVA with Tukey’s post hoc test (**B**,**E**). ^★★^, *p* < 0.01.

Accumulating evidence indicates that BRCA1, a key player in the DNA damage response, maintains genomic stability and suppresses tumorigenesis by enhancing DNA double-strand break repair through homologous recombination (HR) [25]. Therefore, we determined the functional roles of OGE in cisplatin-induced DNA damage. In OGE-untreated (control) HepG2 cells, a weak γH2AX signal was detected after cisplatin (60 μM) treatment for 4 h (defined as 0 h post cisplatin treatment), which then peaked at 2 h post cisplatin treatment. The levels of γH2AX gradually reduced as DNA repair was completed (Figure 5A, upper panel). Notably, OGE pretreatment dramatically increased cisplatin-induced DNA damage and γH2AX levels, and these signals were sustained until 24 h post cisplatin treatment (Figure 5A, lower panel).

Increasing levels of DNA damage can lead to an increase in the number of cells entering permanent arrest, allowing repair machinery to act and fix DNA lesions [26]. Consistent with this, our current findings showed that the combination of cisplatin and OGE treatment significantly arrested the cell cycle at the G0/G1 phase (Figure 5B). With DNA damage, γH2AX accumulates in nuclei foci for the recruitment of repair factors [27]. In line with the Western blotting results (Figure 5A), the confocal microscopy assay showed that stronger γH2AX signals accumulated in the nuclei foci of HepG2 cells with the combination treatment of OGE and cisplatin compared to cisplatin treatment alone (Figure 5C). Additionally, the combination treatment prolonged the recovery time from DNA damage (Figure 5C). RAD51 recombinase, an effector recruited to DNA damage sites, is a critical downstream component in HR-mediated DSB repair [28]. OGE did not significantly abolish the recruitment of RAD51 to DNA damage foci in the nuclei (Figure 5C), which could result from the partial inhibition of BRCA1 by OGE. Therefore, apart from impairing DNA repair mediated via the HR pathway, the adjuvant effects of OGE could synergize with cisplatin to cause serious DNA damage.

To further determine whether OGE sensitizes HCC cells to cisplatin via inhibiting BRCA1, we knocked down BRCA1 (BRCA1/KD) using shRNA in HepG2 cells. Western blotting showed that BRCA1/KD partially suppresses BRCA1, although the inhibitory effect of BRCA1/KD is slightly weaker than that of OGE (Figure 5D). The combination of BRCA1/KD and OGE did not induce more BRCA1 inhibition compared to OGE alone, indicating that OGE directly inhibits BRCA1 expression (Figure 5D). Functional assays revealed that the combination of BRCA1/KD and OGE does not increase the sensitivity of HepG2 cells to cisplatin compared to OGE treatment alone (Figure 5E), consistent with the Western blotting results. In summary, OGE could sensitize HCC cells to cisplatin by directly suppressing BRCA1 expression.

### 2.6. OGE Attenuates the EMT and Stemness Features of HCC Cells

**Figure 6:**
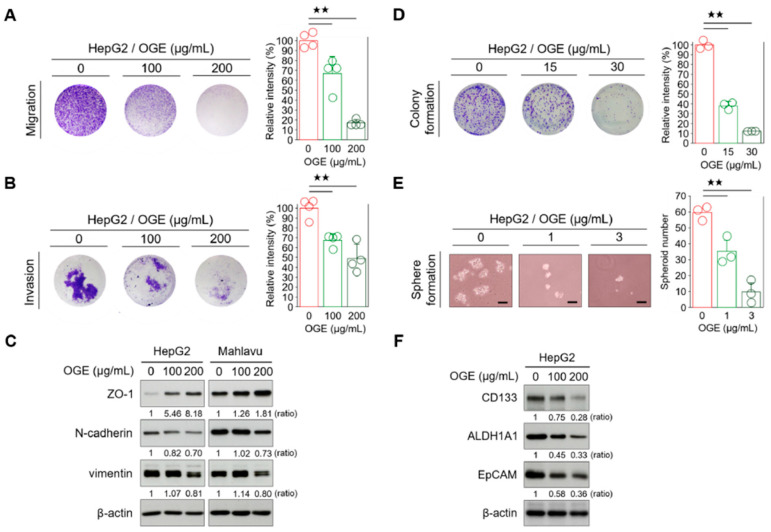
OGE attenuates the EMT and stemness features of HCC cells. (**A**,**B**) Transwell migration (**A**) and invasion assays (**B**) were performed using HepG2 cells treated with the indicated doses of OGE. Signal quantification of crystal violet extracts was measured via colorimetric analysis at 570 nm (*n* = 4). (**C**) The levels of EMT-related proteins in HepG2 and Mahlavu cells were determined via Western blotting. (**D**) A clonogenic assay using HepG2 cells treated with the indicated doses of OGE. Signal quantification was measured by colorimetric analysis at 570 nm (*n* = 3). (**E**) Spheroid formation assay of HepG2 cells treated with the indicated doses of OGE. Scale bar, 50 μm. (**F**) The levels of CSC-related proteins in HepG2 cells. Ratio, the relative protein level. β-actin, loading control. Data are shown as the means ± SD. One-way ANOVA with Tukey’s post hoc test (**A**,**B**,**D**,**E**). ^★★^, *p* < 0.01.

The resistance of cancer cells to therapy is the main cause of death in cancer patients [29]. Both epithelial-to-mesenchymal transition (EMT) and CSC features have been associated with therapy resistance in many cancers [30,31]. In the earlier sections, we found that OGE potentially causes BRCA1 protein instability and proteasomal degradation, thereby sensitizing HCC cells to cisplatin. Given the complex composition of OGE, we wondered if OGE could also impact chemoresistance through the modulation of EMT and stemness in HCC cells. Therefore, we investigated the effects of OGE on the metastatic features of HCC cells. Transwell assays revealed that OGE reduced the migration and invasion abilities of HepG2 cells in a dose-dependent manner (Figure 6A,B). Additionally, OGE increased the expression of the epithelial marker zonula occludens-1 (ZO-1) and decreased the expression of mesenchymal markers N-cadherin and vimentin (Figure 6C), suggesting a potential role of OGE in EMT suppression. Moreover, OGE impaired the colony formation of HepG2 cells (Figure 6D), implying that OGE reduced the capacity for unlimited proliferation of HCC cells [32]. Furthermore, OGE significantly reduced the spheroid formation ability of HepG2 cells in a dose-dependent manner (Figure 6E). Current studies have identified a panel of CSC-specific markers in HCC cells [33]. Consistent with these biological functions, Western blotting verification showed that OGE effectively inhibits the expression of CSC markers in HCC cells, including CD133, aldehyde dehydrogenase (ALDH), and epithelial cell adhesion molecule (EpCAM) (Figure 6F). Taken together, our results demonstrate that OGE can inhibit BRCA1, impair DNA damage repair, and attenuate the EMT and stemness features, thereby reducing chemoresistance in HCC cells.

### 2.7. OGE Enhances the Efficacy of Cisplatin in a Nude Mouse Model of HCC

**Figure 7:**
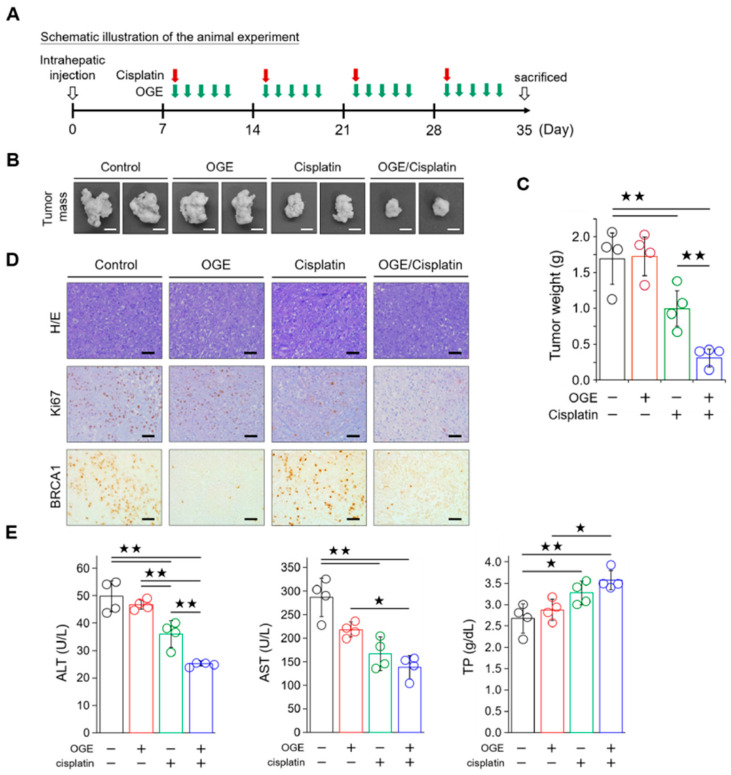
OGE improves the efficacy of cisplatin in mouse models of HepG2 cells. (**A**) Schematic illustration of the animal experiment. (**B**) Representative images showing tumor masses in the livers of mice with the indicated treatments. Scale bar, 0.5 cm. (**C**) Tumor masses after the indicated treatments were weighed and plotted. (**D**) Representative IHC images showing the indicated stains in the formalin-fixed and paraffin-embedded liver tissues. Scale bar, 50 μm. (**E**) The levels of ALT, AST, and TP in blood samples were determined using a serum biochemical analyzer (Autoanalyzer Hitachi 7150) and analyzed using built-in software. Data are shown as the means ± SD. One-way ANOVA with Tukey’s post hoc test (**C**,**E**). ^★^, *p* < 0.05; ^★★^, *p* < 0.01.

To evaluate the efficacy of OGE in sensitizing HCC cells to cisplatin in vivo, HepG2 cells (1 × 10^6^ cells) were orthotopically inoculated into the livers of BALB/c nude mice. A schematic of the animal experiment is shown in Figure 7A. The animals were randomly assigned to four groups: control, OGE alone, cisplatin alone, and a combination of OGE and cisplatin. The treatment regimen consisted of administering cisplatin once a week and OGE for 5 consecutive days, followed by a 2-day rest for a total of four cycles. When administered alone, cisplatin exhibited greater efficacy in reducing tumor growth than OGE, which was ineffective in inhibiting tumor growth. The combination of OGE and cisplatin demonstrated significantly improved efficacy in inhibiting HepG2 cell growth (Figure 7B,C). Consistent with this result, the expression of Ki67, a marker of cell proliferation, in the IHC of the liver tissues revealed a marked decrease in the cisplatin group, particularly in the combination group (Figure 7D). Moreover, OGE effectively reduced BRCA1 levels, which aligns with the mechanistic findings (Figure 7D). Additionally, to understand the effect of the OGE and cisplatin combination on ameliorating liver damage in experimental mice, we conducted serum biochemical tests to assess liver function. Alanine aminotransferase (ALT) and aspartate aminotransferase (AST), found mainly in the liver, are involved in intermediary metabolism and liver gluconeogenesis. High serum levels of ALT and AST usually indicate liver damage [33]. Serum total protein (TP), primarily synthesized in the liver, serves as an index of liver function, with liver dysfunction often decreasing TP levels [34]. The combination of OGE and cisplatin demonstrated the greatest effectiveness in decreasing ALT and AST levels while simultaneously enhancing TP levels (Figure 7E), indicating a potential restoration of liver function. Collectively, these results indicate that OGE has an adjuvant effect on improving cisplatin efficacy for HCC treatment.

## 3. Materials and Methods

### 3.1. Reagents

MTT reagent 3-(4,5-dimethylthiazol-2-yl)-2,5-diphenyltetrazolium bromide (#M6494) was purchased from Invitrogen (Carlsbad, CA, USA); DMSO (#D5879), ethanol (#459836), acetic acid (#A6283), formalin (#15512), crystal violet (#C0775), propidium iodide (#537059), cisplatin (#479306), doxorubicin (#D5220) were purchased from Sigma-Aldrich (St. Louis, MO, USA); PVDF membrane (#88518), cycloheximide (#J66901.03), and RNase A (#12091021) were purchased from Thermo Scientific (Waltham, MA, USA); MG132 (#sc-351846), sorafenib (#sc-220125), lenvatinib (#sc-488530), and gemcitabine (Santa Cruz Biotechnology #sc-481710) were purchased from Santa Cruz Biotechnology (Dallas, TX, USA); chloroquine (#C3730) was obtained from Tokyo Chemical Industry (Tokyo, Japan), DMEM (#12100-046), fetal bovine serum (#26140-079), penicillin/streptomycin (#15070-063), and nonessential amino acids (#11140-050) were purchased from Gibco (Waltham, MA, USA).

### 3.2. O. gratissimum Cultivation and OGE Preparation

The cultivation and experimental research focused on the *medicinal plant O. gratissimum* Linn. complied with the Requirements and Facility Standards for the Qualification of Plant Seed Enterprises set by the Ministry of Agriculture, Executive Yuan, Taiwan. The breeding and propagation of nursery stock was approved by the Executive Yuan Committee on Agriculture, letter no. 0158875A, dated 6 December 1980. The cultivation of *O. gratissimum* Linn. and the collection of experimental leaves (Figure 1A) were conducted at the private farm of Dr. Jer-Yuh Liu (location: Mingjian, Nantou, Taiwan; latitude 23°50′ N 120°42′ E) from August 2020 to March 2023 under the guidelines and legislation of competent authorities. After harvesting, *O. gratissimum* leaves were washed under running water for 10 min, followed by indoor withering for 1 week. The dried leaves were ground into a fine powder using a polytron and then mixed with distilled water at a final concentration of 400 g/L. The homogenate was incubated at 95 °C for 1 h and then filtered through two layers of gauze. The filtrate was centrifuged at 5000× *g* at 4 °C for 3 h to remove insoluble pellets. The supernatant was collected and lyophilized, and the extract powders were stored at −70 °C until use.

### 3.3. Cell Culture

Human hepatoblastoma cell HepG2 (ATCC #HB-8065) was obtained from the American Type Culture Collection (Manassas, VA, USA). Human hepatocarcinoma cell Mahlavu was gifted by Dr. Jaw-Ching Wu from the Institute of Clinical Medicine of National Yang-Ming Chiao-Tung University, Taipei, Taiwan. These cells were maintained in DMEM (Gibco; Waltham, MA, USA) supplemented with 10% fetal bovine serum (Gibco; Waltham, MA, USA), 100 μg/mL penicillin/streptomycin (Gibco; Waltham, MA, USA), and 1% nonessential amino acids (Gibco; Waltham, MA, USA) in a humidified atmosphere containing 5% CO_2_ and 95% air at 37 °C.

### 3.4. Cell Viability Assay

The effects of chemotherapeutic drugs and OGE on cell viability were determined via a methylthiazol tetrazolium (MTT) method. Tumor cells were seeded on 24-well microplates at a density of 2 × 10^4^ cells/well and treated with reagents for 24 h. After treatment, 200 μL MTT solution (1 g/L in PBS) was added at 37 °C for 4 h. The reduced formazan compounds were dissolved using DMSO (Sigma-Aldrich; St. Louis, MO, USA) and measured at a wavelength of 570 nm with an enzyme-linked immunosorbent assay reader (Dynex). Cell viability (%) = (experiment OD570/control OD570) × 100%.

### 3.5. Cell Cycle Analysis

After 24 h of culture in serum-free media, HepG2, in the presence or absence of OGE (200 μg/mL), was treated with cisplatin (60 μM) for 6 h. Then, the tumor cells were harvested and fixed in 70% ethanol (Sigma-Aldrich; St. Louis, MO, USA) at 4 °C. After ethanol removal, and PBS washing, the tumor cells were reacted with RNase A (20 μg/mL; Thermo Scientific; Waltham, MA, USA) at 37 °C for 15 min and stained with propidium iodide (20 μg/mL). The cell cycle distribution was determined using a FACSVerse flow cytometer (BD Biosciences; Franklin Lakes, NJ, USA). The cell distribution at different stages of the cell cycle was analyzed using BD FACSuite software (v.1.0.6; BD Biosciences, Franklin Lakes, NJ, USA).

### 3.6. Transwell Migration and Invasion Assays

For the in vitro migration assay, tumor cells (2 × 10^4^ cells in 200 μL media) were suspended in the upper half of a PET membrane transwell insert chamber (BD Biosciences; Franklin Lakes, NJ, USA) on a 24-well plate. For the in vitro invasion assay, tumor cells (4 × 10^4^ cells in 200 μL media) were suspended in transwell insert chambers coated with Matrigel (1 g/L; BD Biosciences; Franklin Lakes, NJ, USA). Media without FBS supplementation were added into the upper chamber, whereas media with 10% FBS supplementation were added into the lower chamber. After incubation at 37 °C for 24 h (for migration assay) or 48 h (for invasion assay), tumor cells that had passed through the insert were fixed with 3.7% formalin (Sigma-Aldrich; St. Louis, MO, USA) and stained with 0.1% crystal violet (Sigma-Aldrich; St. Louis, MO, USA). After extraction using 50% ethanol and 0.1% acetic acid (Sigma-Aldrich; St. Louis, MO, USA), the crystal violet solution was measured using colorimetry at 570 nm for quantification.

### 3.7. Colony Formation Assay

For the colony formation assay, tumor cells (1000 cells/well) were seeded into 6-well plates and then treated or untreated (control) with indicated doses of OGE. After culturing for 10 d, tumor cells were fixed with 3.7% formalin (Sigma-Aldrich; St. Louis, MO, USA) and stained with 0.1% crystal violet (Sigma-Aldrich; St. Louis, MO, USA). For quantification, the stained dyes were extracted using 50% ethanol (Sigma-Aldrich; St. Louis, MO, USA) and 0.1% acetic acid (Sigma-Aldrich; St. Louis, MO, USA) and measured via colorimetry at 570 nm.

### 3.8. Spheroid Formation Assay

Tumor cells (2000 cells/dish) treated or untreated (control) with indicated doses of OGE were cultured in 6 cm culture dishes coated with 1% agarose for 7 days. The numbers of spheroid formation were counted manually under a bright-field microscope.

### 3.9. Western Blotting Assay

Before blotting, the total proteins of the tumor cells were separated using a 9.5% or 12.5% SDS-PAGE gel based on the molecular weight of the target protein. For Western blotting, proteins were transferred onto a PVDF membrane (Thermo Scientific; Waltham, MA, USA) at 400 mA at 0 °C for 3 h in a transfer buffer containing 25 mM Tris-HCl, 197 mM glycine, and 13.3% (*v*/*v*) methanol. Membranes were blocked with 5% (*w*/*v*) skim milk in TBST for 1 h and then incubated with primary antibodies at 4 °C for 16–24 h. The primary antibodies used in this study are summarized in Supplementary Table S2. The horseradish peroxidase-conjugated secondary antibody was incubated with the membrane at room temperature for 1 h. After washing, immunoreactive signals were revealed using an enhanced ECL substrate Western Lighting Plus-ECL (PerkinElmer #PK-NEL105; Shelton, CT, USA) and recorded by developing photographic film under optimum exposure conditions or using the luminescence image analyzer ImageQuant LAS 4000 (GE Healthcare Life Sciences; Washington, DC, USA). The original images of the Western blot assays are shown in Supplementary Figure S1. For relative quantification, the protein intensity was analyzed using ImageJ 1.54g software. The relative target protein level (target/actin) in each group was normalized to the control.

### 3.10. Pulse-Chase Assay

Pulse-chase analysis is a commonly used technique for studying the synthesis, processing, and transport of proteins. In this study, cycloheximide (CHX, 10 g/L) was used to inhibit newly synthesized BRCA1 proteins 1 h before OGE (200 μg/mL) treatment (“pulse”). Compared to the control (without OGE treatment), the effect of OGE on enhancing BRCA1 degradation can be resolved via Western blotting validation (“chase”).

### 3.11. Quantitative Polymerase Chain Reaction (qPCR)

The total RNAs of the tumor cells with or without OGE treatment were extracted using TRIzol reagent (Invitrogen #15596018; Carlsbad, CA, USA). The cDNA was generated via reverse transcription PCR using MMLV first-strand synthesis kits (GeneDireX #MB301-0050; Taoyuan, Taiwan). The dilution products were reacted with KAPA SYBR FAST qPCR Master Mix (2X) Kit (Kapa Biosystems #KR0389; Wilmington, MA, USA) and subjected to qPCR analysis using the LightCycler 480 apparatus (Roche; Basel, Switzerland). Individual GAPDH, β-actin, and 18S rRNA served as the endogenous control. The sequences of qPCR primers are summarized in Supplementary Table S3. The mRNA expression levels were calculated via a comparative Ct method using 2^−ΔΔCt^.

### 3.12. Proteomic Identification

Proteomic alterations in HepG2 cells treated with OGE (100 μg/mL or 200 μg/mL) were analyzed via mass spectrometric analysis (MS). The total proteins of the tumor cells were extracted via the RIPA Lysis and Extraction Buffer (Thermo Scientific #89900; Waltham, MA, USA) combined with sonication. Protein concentrations were measured using the Bio-Rad Protein Assay kit (#5000006EDU) at 595 nm absorbance. The total proteins (20 μg) were separated using a 9.5% SDS-PAGE gel and divided into five gel fractions for tryptic peptide preparation using an in-gel digestion method. The MS method referred to our previous report [35]. An Orbitrap Exploris 480 mass spectrometer (Thermo Scientific; San Jose, CA, USA) equipped with an Ultimate 3000 RSLC system (Thermo Scientific; San Jose, CA, USA) and a nano-electrospray ion source (Thermo Scientific; San Jose, CA, USA) was used for MS analysis. The MS instrument was operated in the positive ion mode with a spray voltage set to 1.85 kV in the data-dependent acquisition mode. The survey scan was set at a mass range of *m*/*z* 375–1500 (AGC target, 4 × 10^5^) and a resolution of 120,000 at *m*/*z* 200. The twenty most abundant multiple-charged ions were sequentially fragmented via collision-induced dissociation for tandem mass analysis in the Orbitrap at a resolution of 30,000. Protein identification and label-free quantification were performed using Proteome Discovery software (v2.4). The identification threshold was set at a *p*-value < 0.05.

### 3.13. In Silica Survival Analysis

The effect of BRCA1 on overall survival (OS) and progression-free survival (PFS) of HCC patients was evaluated by the Kaplan-Meier plotter server (http://kmplot.com/analysis/, accessed on 6 June 2023), which contained independent datasets from the Cancer Biomedical Informatics Grid (caBIG), the Gene Expression Omnibus (GEO), and the Cancer Genome Atlas (TCGA) repositories. The high versus low expression levels of BRCA1 mRNA were split by the median value. The threshold of follow-up of patients was set as 60 months. The hazard ratio (HR) was given with 95% confidence intervals, and the log-rank *p*-value was calculated and displayed on the webpage.

### 3.14. Mouse Orthotopic Tumor Model and Antitumor Assay

The animal study protocol (CMUIACUC-2018-196) was reviewed and approved by the Institutional Animal Care and Use Committee at China Medical University Hospital. All methods followed relevant guidelines and regulations, and data were reported based on ARRIVE guidelines (https://arriveguidelines.org, accessed on 1 May 2022) [36]. The animal experiments were conducted at the Animal Center on the 10th floor of the Cancer Center Building, China Medical University Hospital. Mice were anesthetized with 25 mg/kg of Zoletil 50 and 10 mg/kg of Rompun via intraperitoneal injection. An orthotopic mouse model of HCC cells was followed by injecting HepG2 cells (1 × 10^6^ cells/30 μL serum-free DMEM) into the liver of 6-week-old male BALB/c nude mice (BALB/cAnN.Cg-*Foxn1^nu^*/CrlNarl) using a syringe with a 29-gauge needle (defined as day 0). One week after inoculation, mice were randomly assigned into four groups for treatment: (1) the control group (saline treatment, *n* = 4); (2) the OGE group (*n* = 4); (3) the cisplatin group (*n* = 4); and (4) combination of OGE and cisplatin group (*n* = 4). For one treatment cycle, cisplatin 1 time/week (10 mg/kg, intraperitoneal injection) and OGE 5 times/week (40 mg/kg, oral administration) were given, and a total of 4 treatment cycles were performed. The animals were sacrificed on day 35, and tumor masses were weighed and fixed in formalin for immunohistochemistry (IHC) analysis. The levels of alanine aminotransferase (ALT), aspartate aminotransferase (AST), and serum total protein (TP) in the blood samples were determined through the use of a serum biochemical analyzer (Autoanalyzer Hitachi 7150; Tokyo, Japan) using analysis kits (Point Scientific; Canton, MI, USA).

### 3.15. Statistics

The quantitative characteristics of the data were displayed as mean and standard deviation (SD). Whether the difference between the responses of the two groups is statistically significant was analyzed by a two-tailed Student’s *t*-test, which was used for MTT assay and qPCR assay. One-way ANOVA followed by Tukey’s post hoc test was used for the comparison of multiple groups, such as MTT assay, cell migration, invasion, and spheroid formation assay, tumor masses comparison, and biochemical tests of liver function. OS and PFS were determined via the Kaplan-Meier method. Survival curves were compared using the log-rank test. Statistical analyses were performed using IBM SPSS Statistics 22. The significance level was set as 0.05.

### 3.16. Data Availability

All of the MS raw datasets in this study were deposited in jPOST [37] and ProteomeXchange. The accession numbers are JPST003112 for jPOST and PXD052281 for ProteomeXchange. The data can be accessed on 15 May 2024 through https://repository.jpostdb.org/preview/53147652366459319cd33c3 with access key 1714.

## 4. Discussion

In recent decades, targeting therapies have been recommended for patients with HCC worldwide. Sorafenib, a multiple tyrosine kinase inhibitor (TKI), showed a median OS of 10.7 months compared to 7.9 months in the placebo group (hazard ratio [HR], 0.69; 95% confidence interval [CI], 0.55–0.87; *p* < 0.001) in the SHARP study and with a median OS of 6.5 months compared to 4.2 months in the placebo group (HR, 0.68; 95% CI, 0.50–0.93; *p* = 0.014) in an Asia-Pacific region study. Sorafenib was approved as the first-line systemic treatment for advanced HCC [38,39]. Furthermore, lenvatinib, a TKI targeting VEGFR, PDGFRα, KIT, and RET, with a median OS of 13.6 months, was also approved as a first-line treatment for unresectable HCC [40]. In the phase III HIMALAYA trial, a single priming dose of tremelimumab plus once-monthly durvalumab demonstrated better OS compared to sorafenib as a first-line treatment for patients with inoperable HCC [41].

Recently, regorafenib, cabozantinib, and ramucirumab were successfully approved as second-line treatments for patients with HCC who experienced disease progression after receiving first-line sorafenib treatment [42,43,44]. In addition, immune checkpoint inhibitors nivolumab and pembrolizumab have been shown to improve OS in patients with advanced HCC who had previously received sorafenib, leading to approval by the FDA as second-line treatments for advanced HCC [45,46]. However, immunotherapy alone has limited antitumor efficacy in terms of enhancing OS compared to sorafenib in treatment naïve patients, indicating a necessity for reliable biomarkers for precision medicine. Other chemotherapeutic agents, such as doxorubicin, 5-fluorouracil, and cisplatin, have proven effective in HCC treatment, but only a small proportion of patients can benefit from these treatments. Systemic chemotherapy usually offers a modest benefit in terms of OS, disease-free survival, and disease control rate, but it is accompanied by a significant percentage of adverse events. Cisplatin, a platinum-based chemotherapy drug, exerts a cytotoxic effect by inducing DNA damage and impairing DNA replication and transcription. It is broadly used to treat various cancers, including head and neck squamous cell carcinoma, esophageal cancer, lung cancer, ovarian cancer, bladder cancer, and cervical cancer. To enhance drug concentration within the intrahepatic circulation and avoid systemic accumulation, cisplatin combined with other chemotherapy drugs using hepatic arterial infusion has been evaluated in recent clinical trials [47,48]. Liver functional failure is one of the leading causes of therapeutic limitation and cancer-related death. Additionally, HCC cells exhibit significant chemotherapy-refractory characteristics, which pose a crucial obstacle to treatment response [7]. Consequently, developing chemosensitizers could be an important approach to maximizing the applications of chemotherapy agents for HCC treatment.

Natural products can be used for therapeutic purposes and serve as phytonutrients in health supplements [49]. It is well known that dietary habits are associated with the prevention and treatment of cancers; for instance, a higher dietary intake of fruits and vegetables is associated with a lower risk of cancer development [50]. The compounds in natural products could activate anti-inflammatory, antioxidant, and antitumor mechanisms, potentially providing therapeutic options for new cancer treatment regimens [51]. *O. gratissimum*, commonly known as African basil or clove basil, is a medicinal plant with various therapeutic properties. Traditionally, *O. gratissimum* has been used for the treatment of cough, fever, inflammation, diarrhea, anemia, and snakebites, and it has also been used as a mosquito repellent [52]. Due to its commercial availability, tea products of *O. gratissimum* can be utilized as dietary supplements or for clinical assessment to evaluate their chemosensitizer activities. The phytochemical components of *O. gratissimum* include alkaloids, saponins, tannins, phlobatannins, glycosides, phenols, anthraquinones, flavonoids, and terpenoids [53]. However, the genetic background and growth environment of the plants could affect the phytochemical components and influence therapeutic effectiveness [54]. Therefore, we determined the composition profile of OGE using an accurate and sensitive HPLC-MS technique, establishing it as a standardized reference (Figure 1). Among the identified components, caffeic acid, caftaric acid, and rosmarinic acid are commonly reported in studies of *Ocimum* species [55]. In this study, we showed that OGE enhanced the sensitivity of HCC cells to cisplatin by amplifying the DNA damage effect caused by cisplatin (Figure 5 and Figure 7). However, the active compounds in OGE that contribute to this effect need to be further characterized.

Although combinations of herbal products have been evaluated to enhance cisplatin-associated chemotherapy against various cancers in clinical trials (Trial ID: NCT01975454, NCT01441752, and NCT02638051), clinical evidence of the adjuvant efficacy of single herbal medicine remains scarce [12]. Several herbal medicines have been reported as chemosensitizers that promote cisplatin efficacy. For instance, hibiscus extracts increased oxidative stress and induced apoptosis synergistically with cisplatin in triple-negative breast cancer [56]. *Ephedra alata* extracts induced apoptosis in a caspase-dependent manner and exhibited synergic anti-proliferative and pro-apoptotic actions with cisplatin [57]. *Juniperus indica Bertol* extracts induced melanoma cell apoptosis and synergized with cisplatin by suppressing the AKT/mTOR and MAPK pathways [58]. Hydroethanolic extracts from *Pouteria ramiflora* leaves increased cisplatin-induced cytotoxicity and apoptosis in HCC cells [59]. In this study, OGE was identified as a potential chemosensitizer for HCC treatment. The correlation between the stemness and metastatic properties of tumor cells and treatment resistance is well recognized [31,60]. Surprisingly, knowledge regarding the impact of *Ocimum* species on these properties is quite limited. A recent study reported that basil polysaccharide alleviates tumor hypoxia to inhibit HCC cell metastasis and progression through the suppression of hypoxia-inducible factor 1α-mediated EMT [61]. Our current results revealed the versatile functions of OGE in suppressing HCC cell progression, such as impairing migration and invasion features, reprogramming EMT, and attenuating CSC properties (Figure 6). The comprehensive effects likely result from the multiple constituents of OGE, highlighting the possible advantages of utilizing herbal medicine. This finding may broaden our understanding of the roles of OGE and expand its potential applications.

Therapeutic drugs are developed to target different signaling pathways for tumor control. Doxorubicin intercalates into DNA and inhibits topoisomerase II, thereby preventing the recombination of DNA double-strand and stopping DNA replication [62]. Sorafenib is an oral multiple kinase inhibitor that suppresses tumor proliferation through the RAF/MEK/ERK pathway and reduces angiogenesis by inhibiting VEGFR and PDGFR signaling [21]. Lenvatinib is another multiple kinase inhibitor that suppresses proteins involved in the formation of new blood vessels both inside and outside tumors [63]. Gemcitabine is a nucleoside analog that promotes apoptosis in tumor cells undergoing DNA synthesis and blocks tumor progression at the G1/S-phase boundary [64]. Our proteomic analysis and Western blotting validation indicated that OGE sensitized HCC cells to cisplatin by inhibiting BRCA1 (Figure 3 and Figure 4). Consistent with these findings, this mechanism shows greater specificity and primarily enhances the function of cisplatin rather than other drugs tested in this study (Figure 2C–F).

A literature review shows that several herbal compounds have been identified in sensitizing HCC cells to chemotherapy. *Gynura divaricate* combined with cisplatin attenuates tumor growth and relapse in HCC cells by suppressing cancer stem cell growth and the Wnt/β-catenin signaling pathway [65]. Kanglaite injection enhances the efficacy of cisplatin in suppressing HCC cells by inhibiting the CKLF1-mediated NF-κB pathway and regulating transporter-mediated drug efflux [66]. Additionally, dihydrotanshinone I target estrogen receptor α upregulates its expression in a concentration-dependent manner, leading to the downregulation of BRCA1 and resulting in DNA double-strand breaks and proliferation inhibition in HCC cells [67]. In this study, a major difference from previous research is that we adopted a non-biased screening to analyze the sensitizing effect of OGE on commonly used chemotherapy drugs for HCC cell treatment, thereby identifying cisplatin as having the best sensitizing effect by OGE (Figure 2). Moreover, a non-biased proteomic analysis was used to identify the potential target proteins and uncover the mechanism of OGE in sensitizing HCC cells to cisplatin (Figure 3). This non-biased approach allows us to more objectively evaluate the application value of OGE in the treatment of HCC cells. Compared to previous research models, our use of herbal ingredients has its advantages, such as low toxicity and suitability for long-term consumption. However, this advantage also presents some research limitations, such as the difficulty in fully and precisely evaluating the molecular regulatory mechanisms due to its complex molecular composition. Additionally, the genetic background and growth environment of the plants can affect the phytochemical components and influence therapeutic effectiveness [53]. Accordingly, a standardized reference is necessary for the evaluation of OGE bioactivities (Figure 1). Although the single molecules identified in previous research have been validated for their efficacy in vitro and in vivo experiments, regulatory restrictions prevent their use in clinical trials until they have undergone rigorous human testing. As a health drink, the safety of OGE has been verified through long-term consumption and its commercial availability. This is advantageous for the evaluation and testing of our research findings in clinical applications.

BRCA1, a tumor suppressor, is involved in multiple biological processes, including transcription, cell cycle progression, and DNA damage repair [22]. In normal cells, mutations or defects in genes related to DNA repair, such as BRCA1, are well known to be associated with a high risk of cancer. The lifetime risk of breast cancer and ovarian cancer with BRCA1 mutation is approximately 60% (95% CI: 44–75%) and 59% (95% CI: 43–76%), respectively [22]. Conversely, tumors can elicit a DNA damage response to activate DNA repair and cell cycle checkpoints, thereby promoting therapeutic resistance and cell survival [68]. Thus, targeting the DNA damage repair system is regarded as a potential therapeutic strategy, and several candidate molecules are currently under evaluation in preclinical and clinical trials [69]. Because tumors can use an alternate pathway to compensate for the defects of DNA repair genes, precise therapeutic agents against the auxiliary pathway may achieve better therapeutic efficacy through synthetic lethality [70]. PARP inhibitors used for BRCA1/2 mutant tumors, and DNA-PK inhibitors used for ATM-defective tumors are being developed based on this conceptual framework [71].

The mutation prevalence of BRCA1 is generally low, being approximately only 7% in patients with non-familial breast cancer or ovarian cancer [60]. Physiologically, BRCA1 levels fluctuate in a cell cycle-dependent manner. BRCA1 is phosphorylated and accumulates in the nucleus when cells enter the S phase, maintaining stability during mitosis. As cells transition into the G1 phase, BRCA1 undergoes ubiquitination and proteasome-dependent degradation [72]. Additionally, BRCA1 can be degraded via various ubiquitin-proteasome-mediated pathways, including HERC2, HUWE1, FBXO44, and Parkin. These studies suggest an important role of the ubiquitin-proteasome pathway in regulating BRCA1 stability. Consistent with these findings, our study showed that OGE induced BRCA1 instability through the activation of proteasomal degradation (Figure 4C). Therefore, OGE could serve as an effective regimen for targeting wild-type BRCA1, providing an opportunity for combination with PARP inhibitors, such as olaparib, niraparib, and rucaparib, to improve therapeutic outcomes in patients with HCC.

## 5. Conclusions

**Figure 8:**
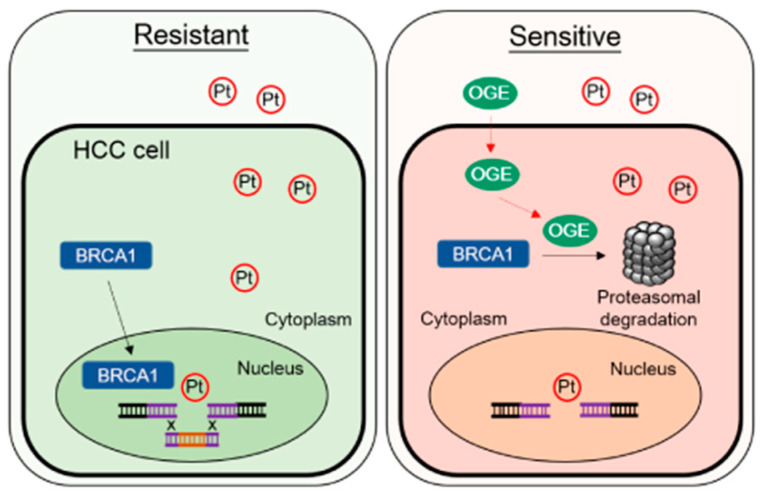
Representative working model. OGE synergistically increases cisplatin-induced DNA damage by activating proteasomal degradation of BRCA1. Abbreviations/symbols: OGE, aqueous extract of *O. gratissimum*; Pt, cisplatin; BRCA1, breast cancer type 1 susceptibility protein; HCC, hepatocellular carcinoma; Resistant, cisplatin-resistant HCC cells; Sensitive, cisplatin sensitive HCC cells.

We demonstrate that OGE effectively inhibits cell migration and invasion abilities, modulates the EMT feature, and impairs stemness properties in HCC cells. Importantly, OGE could serve as a chemosensitizer to improve cisplatin efficacy for HCC treatment both in vitro and in vivo. OGE synergistically increases cisplatin-induced DNA damage, likely via the activation of the proteasomal degradation of BRCA1. The working model of this study is shown in Figure 8.

## Figures and Tables

**Table 1:** Proteins with significant down-regulation by OGE treatment.

UniPort	Gene Name	Protein Name	Abundance Ratio [OGE100]/[CTL]	Abundance Ratio [OGE200]/[CTL]
Q86V13	IQGAP3	Ras GTPase-activating-like protein IQGAP3	0.01	0.01
Q14117	DPYS	Dihydropyrimidinase	0.01	0.01
P45983	MAPK8	Mitogen-activated protein kinase 8	0.01	0.01
QSNA72	POC5	Centrosomal protein POC5	0.01	0.01
Q6VMQ6	ATF7IP	Activating transcription factor 7-interacting protein 1	0.01	0.01
Q3MIP1	ITPRIPL2	Inositol 1,4,5-trisphosphate receptor-interacting protein-like 2	0.01	0.01
Q9BRS8	LARP6	La-related protein 6	0.01	0.01
Q96RQ3	MCCC1	Methylcrotonoyl-CoA carboxylase subunit alpha, mitochondrial	0.01	0.01
Q99081	TCF12	Transcription factor 12	0.01	0.01
Q13796	SHROOM2	Protein Shroom2	0.01	0.01
Q69YH5	CDCA2	Cell division cycle-associated protein 2	0.01	0.01
P38398	BRCA1	Breast cancer type 1 susceptibility protein	0.01	0.01
QSIXM6	NRM	Nurim	0.01	0.01
QSN8A2	ANKRD44	Serine/threonine-protein phosphatase 6 regulatory ankyrin repeat subunit B Homeobox protein SIX4	0.01	0.01
Q9UIU6	SIX4	Homeobox protein SIX4	0.01	0.01
Q13887	KLF5	Krueppel-like factor 5	0.01	0.01
Q6PEV8	FAM199X	Protein FAM199X	0.01	0.01
P21741	MDK	Midkine	0.28	0.01
Q6ZRY4	RBPMS2	RNA-binding protein with multiple splicing 2	0.37	0.12
O00212	RHOD	Rho-related GTP-binding protein RhoD	0.38	0.29
Q8N4T8	CBR4	3-oxoacyl-[acyl-carrier-protein] reductase	0.39	0.17

## Data Availability

All MS raw datasets in this study were deposited in jPOST and ProteomeXchange. The accession numbers are JPST003112 for jPOST and PXD052281 for ProteomeXchange. The data are accessed on 15 May 2024 through the web address https://repository.jpostdb.org/preview/53147652366459319cd33c3 with access key 1714.

## Supplementary Materials

The following supporting information can be downloaded at: https://www.mdpi.com/article/10.3390/ijms25158424/s1.
