# Supplementary material for: Aqueous Extracts of Ocimum gratissimum Sensitize Hepatocellular Carcinoma Cells to Cisplatin through BRCA1 Inhibition
Source: Int J Mol Sci. 2024 Aug 1;25(15):8424. doi: 10.3390/ijms25158424 (PMC11313253; doi:10.3390/ijms25158424)
Supplement: Supplementary file 1 [file ijms-25-08424-s001.zip › ijms-3084214-supplementary figure.pdf]

# Aqueous Extracts of *Ocimum gratissimum* Sensitize Hepatocellular Carcinoma Cells to Cisplatin through BRCA1 Inhibition

Jing-Huei Chen, Tsai-Hui Lin, Yu-Chuan Chien, Chung-Yu Chen, Chih-Tung Lin, Wei-Wen Kuo and Wei-Chao Chang

**Supplementary Figure S1** The original images of Western blotting

Fig. 2A

Cropped blots in main paper    Original blots in supplementary information

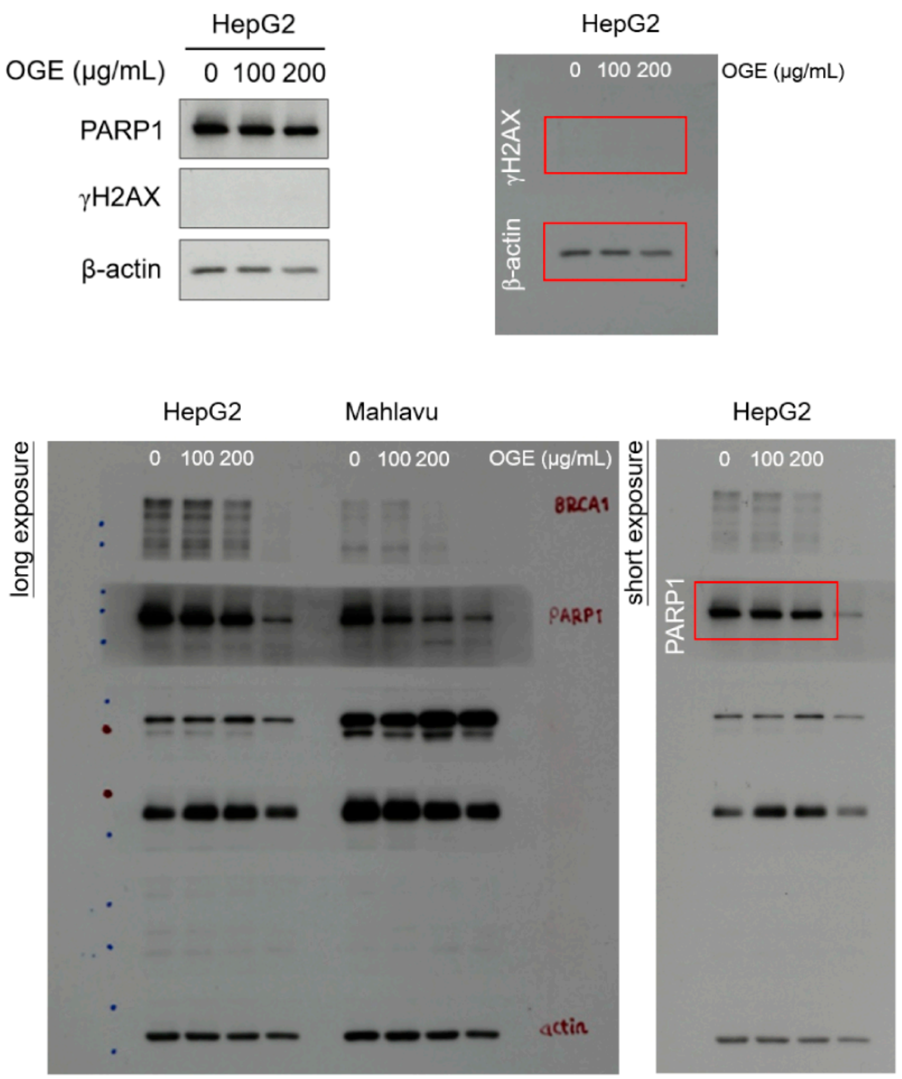

Fig. 3D

Cropped blots in main paper

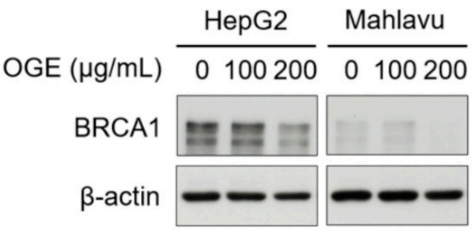


Original blots in supplementary information

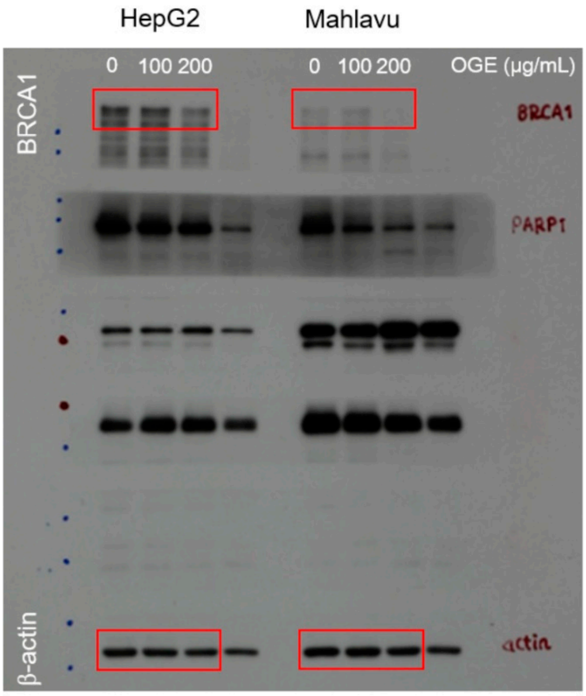

Fig. 4B

Cropped blots in main paper

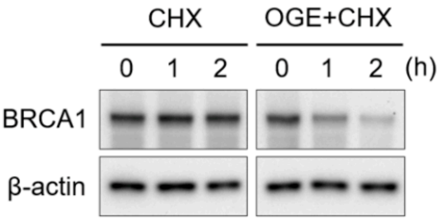


Original blots in supplementary information

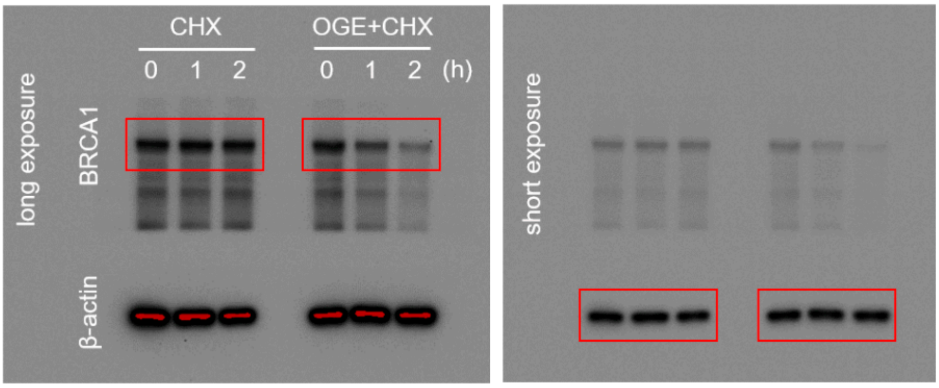

Fig. 4C

Cropped blots in main paper

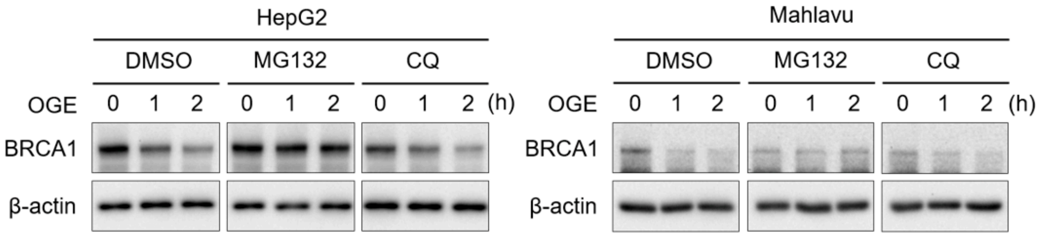


Original blots in supplementary information

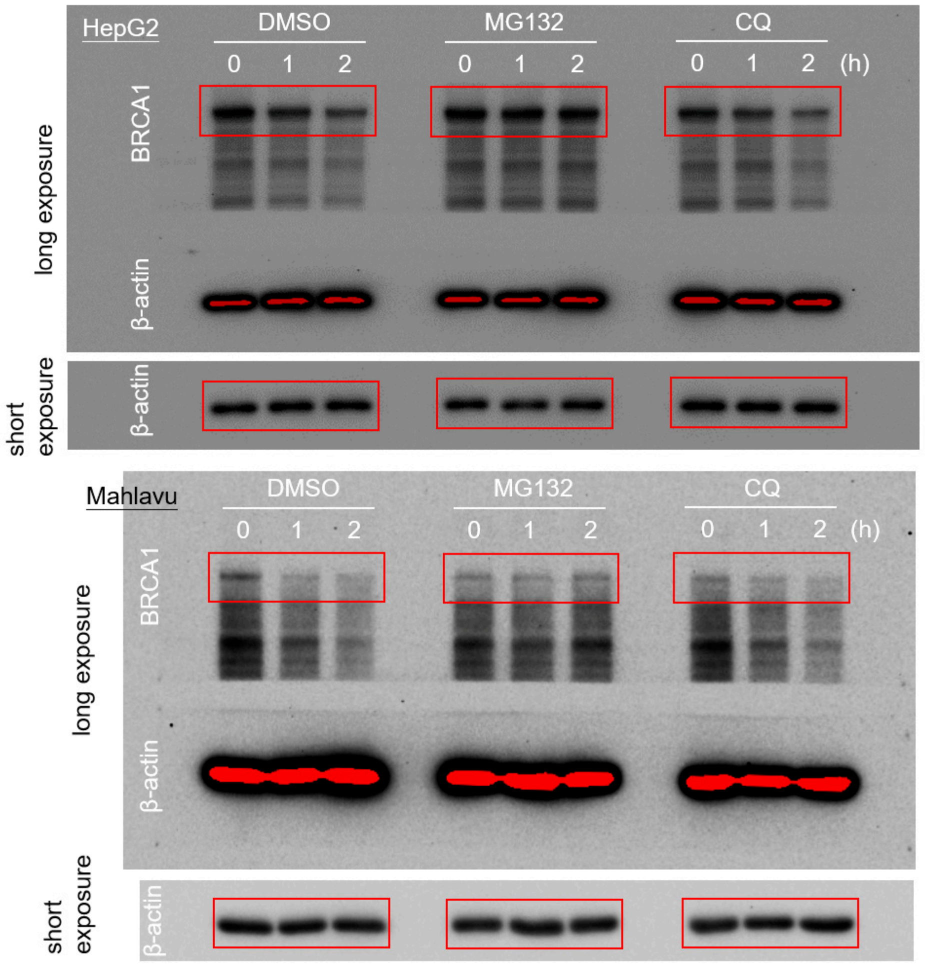

Fig. 5A

## Cropped blots in main paper

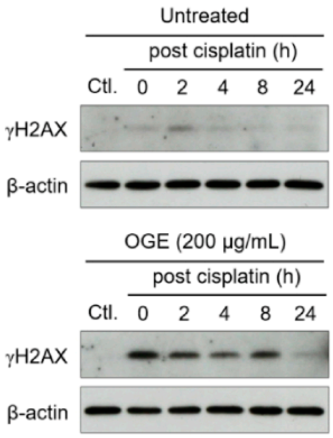


## Original blots in supplementary information

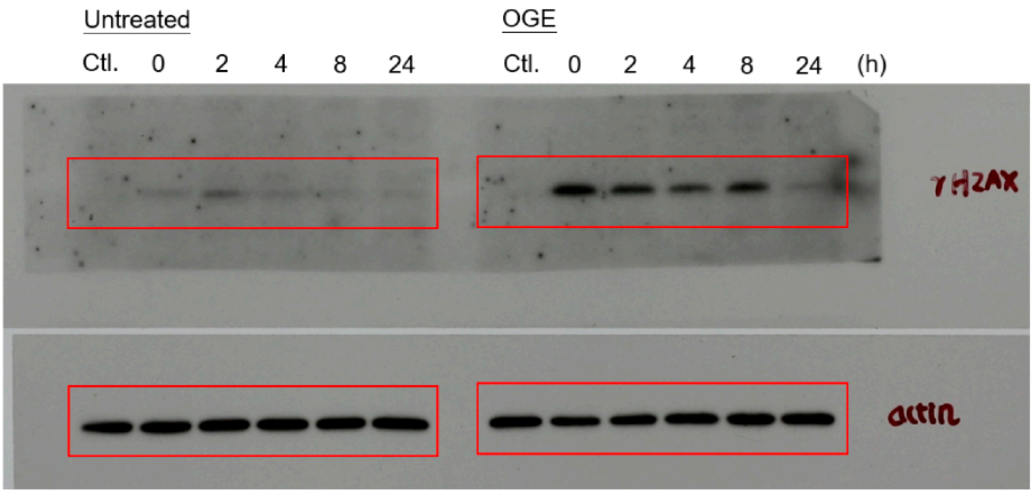

Fig. 5D

Cropped blots in main paper

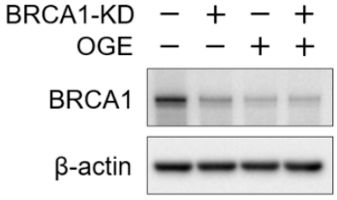


Original blots in supplementary information

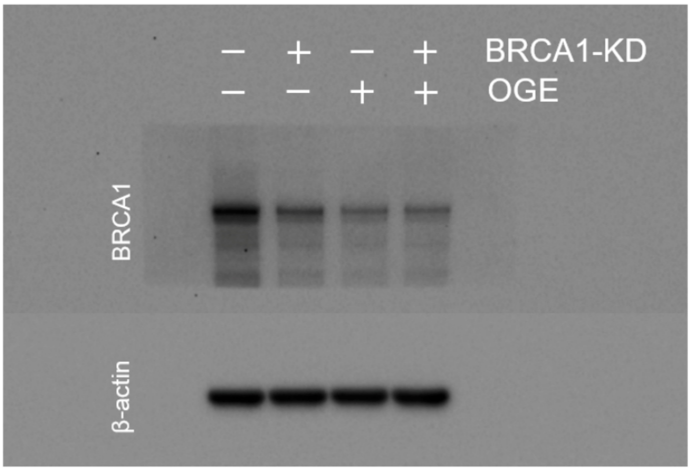

Fig. 6C

Cropped blots in main paper

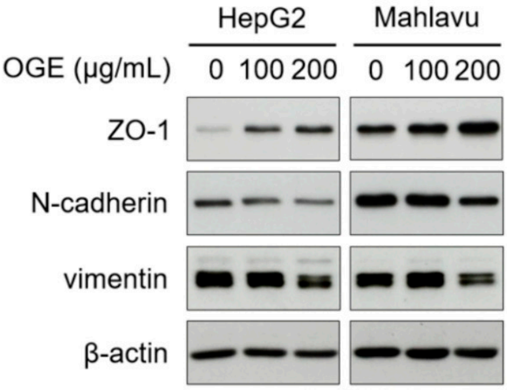


Original blots in supplementary information

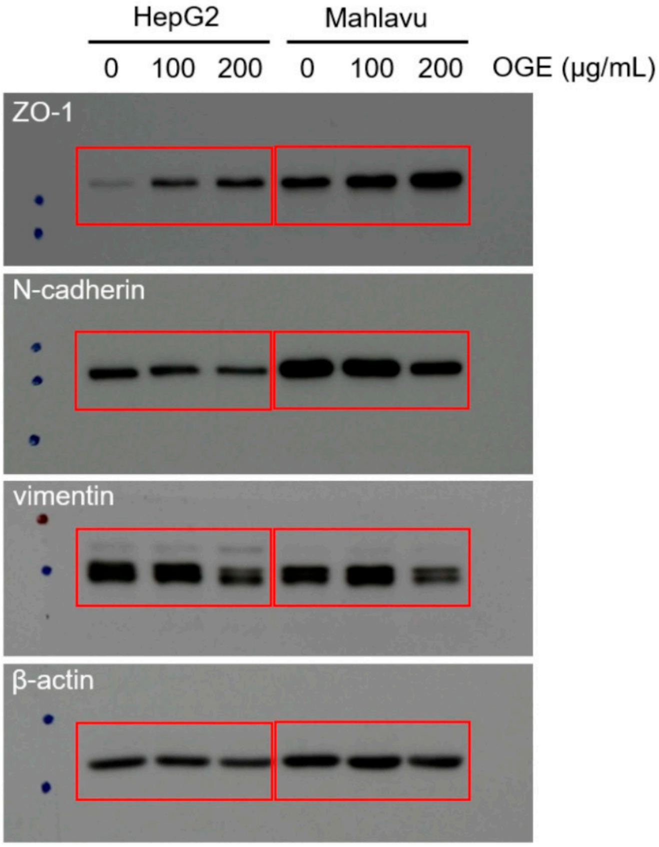

Fig. 6F

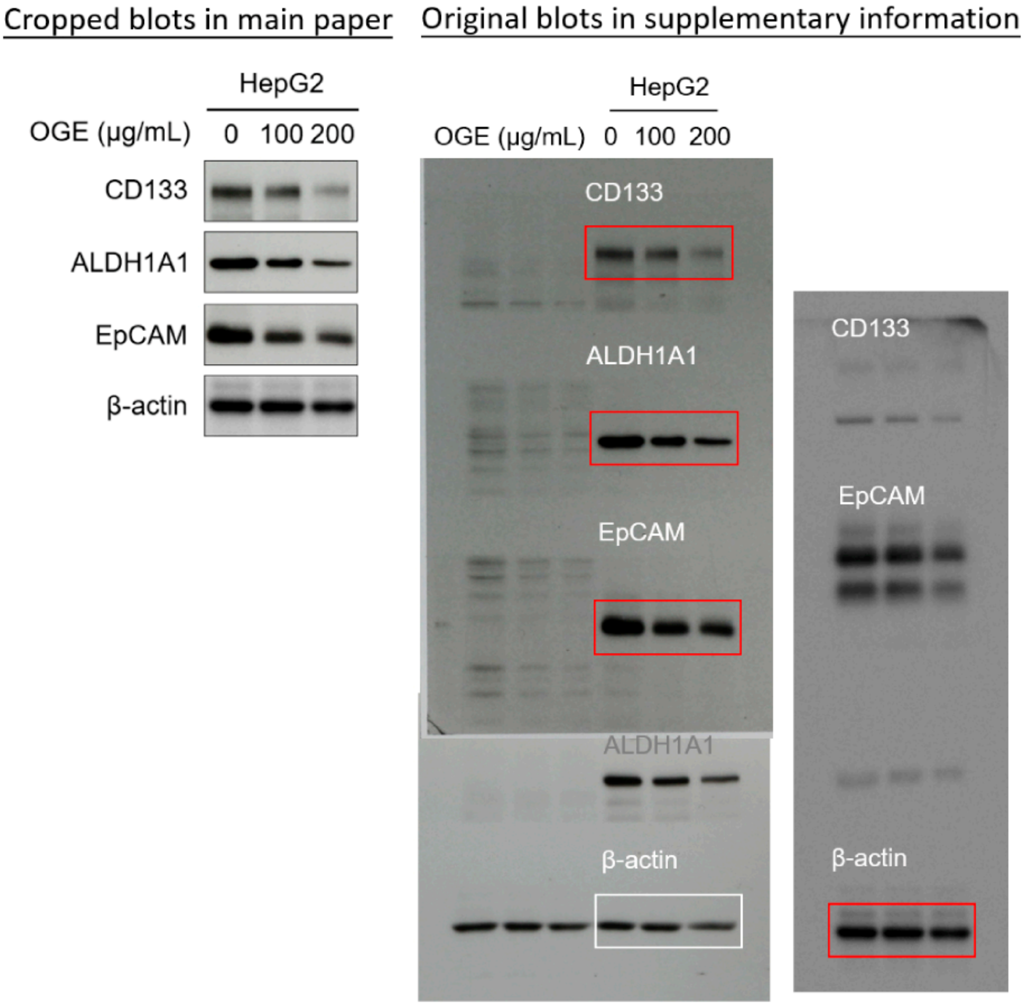
Cropped blots in main paper
Original blots in supplementary information
HepG2
OGE (μg/mL)
0
100
200
CD133
ALDH1A1
EpCAM
β-actin
HepG2
OGE (μg/mL)
0
100
200
CD133
ALDH1A1
EpCAM
ALDH1A1
β-actin
CD133
EpCAM
β-actin
